# Genomic analysis of heat-shock factor targets in *Drosophila*

**DOI:** 10.1186/gb-2005-6-7-r63

**Published:** 2005-06-10

**Authors:** Ian Birch-Machin, Shan Gao, David Huen, Richard McGirr, Robert AH White, Steven Russell

**Affiliations:** 1 Department of Anatomy, University of Cambridge, Downing Street, Cambridge, CB2 3EH, UK; 2 Department of Genetics, University of Cambridge, Downing Street, Cambridge, CB2 3EH, UK

## Abstract

We have used a chromatin immunoprecipitation-microarray (ChIP-array) approach to investigate the *in vivo *targets of heat-shock factor (Hsf) in *Drosophila *embryos. We show that this method identifies Hsf target sites with high fidelity and resolution. Using cDNA arrays in a genomic search for Hsf targets, we identified 141 genes with highly significant ChIP enrichment. This study firmly establishes the potential of ChIP-array for whole-genome transcription factor target mapping *in vivo *using intact whole organisms.

## Abstract 2

A ChIP-assay approach is used for the whole-genome transcription factor target mapping *in vivo* using an intact whole organism. Using this ChIP-array 141 genes are identified in a genomic search for Hsf targets in *Drosophila*.

## Background

Chromatin immunoprecipitation or, more correctly, immunopurification (ChIP) has emerged as a valuable approach for identifying the *in vivo *binding sites of transcription factors [1-6]. Before the availability of complete genome sequence the use of this approach for identifying transcription targets on a genome-wide scale was, however, limited. Over the past few years, a number of laboratories have successfully used high-density DNA microarrays to identify sequences enriched by chromatin immunopurification (the ChIP-array approach). In the yeast *Saccharomyces cerevisiae*, microarrays containing virtually all of the intergenic sequences from the genome have been used to identify the binding sites of a large number of transcription factors [7,8]. In principle, the same techniques can be applied to higher eukaryotes, but the complexity of their genomes presents a challenge for the construction of full genomic microarrays.

Despite such difficulties, several studies have shown the feasibility of the ChIP-array approach with small regions of complex eukaryotic genomes using tissue culture systems. In cultured mammalian cells, for example, the binding sites for several transcription factors have been mapped using microarrays composed of specific promoter regions or enriched for promoter sequences with CpG arrays [9-11]. Although such studies are valuable in identifying some of the targets of particular transcription factors, they are limited because the microarray designs restrict the analysis to proximal promoter elements of a subset of genes. It would be preferable to examine binding sites in an unbiased fashion by constructing tiling arrays composed of all possible binding targets. Such tiling arrays have been constructed on a small scale with microarrays containing a series of 1-kb fragments from the β-globin locus [12], or on a large scale with oligonucleotide arrays containing elements that detect all the unique sequences of human chromosomes 21 and 22 [13]. These studies indicate that the DNA-binding patterns of regulatory molecules in large eukaryotic genomes are complex and highlight the need for a comprehensive approach to understand how transcription factors interact with DNA *in vivo*.

*Drosophila melanogaster*, with a genome complexity intermediate between that of yeast and human, provides a powerful system for investigating transcription factor targets and regulatory networks in a complex multicellular eukaryote. Recently, the principle of using *Drosophila *genome tile arrays to identify transcription factor binding sites in tissue culture cells has been demonstrated. Using a technique employing fusions between DNA-binding proteins and the *Escherichia coli *DNA adenine methyltransferase (DamID; [14]) the binding locations for the GAGA transcription factor and the heterochromatin protein HP1 were mapped within a 3-Mb region of the *Drosophila *genome in a tissue culture system [15]. Other studies have used this method to map proximal binding sites with cDNA arrays [16]. While this elegant technique has the advantage that high-quality antibodies against particular transcription factors are not required, and a recent study indicates that it may be possible to transfer from a tissue culture system to the intact organism [17], it clearly has limitations, as *in vivo *the DAM-tagged transcription factor is not expressed in its normal developmental context. It is therefore desirable to develop methods that allow the mapping of native transcription factors in their correct *in vivo *context within the organism.

Here we adapt chromatin immunopurification techniques using intact *Drosophila *embryos and demonstrate the reliable identification of *in vivo *binding sites for the heat-shock transcription factor Hsf on both genome tile and cDNA arrays. The response of most organisms to heat stress involves the rapid induction of a set of heat-shock proteins (Hsps), including several chaperone molecules that assist in protecting the cell from the deleterious effects of heat [18-21]. Several direct targets of the Hsf transcription factor are already well characterized. In higher eukaryotes, including *Drosophila *and mammals, heat stress results in the trimerization of Hsf monomers, which then bind with high affinity to regulatory elements (heat-shock elements, HSE) close to the transcriptional start sites of *Hsp *genes [22,23]. The *Drosophila *heat-shock system has been characterized at several levels, from the cytological mapping of Hsf-binding sites on polytene chromosomes [22] to the detailed molecular and biochemical analysis of transcriptional regulation at individual *Hsp *genes [24-26]. In this study we extend the analysis of the *Drosophila *heat-shock response by demonstrating that chromatin immunopurification from embryos can accurately map *in vivo *Hsf-binding sites on genome tile microarrays and identify new potential *in vivo *HSEs. In addition, using microarrays containing full-length cDNA clones for over 5,000 *Drosophila *genes we identify almost 200 genes that are reproducibly bound by Hsf upon heat shock in *Drosophila *embryos. The targets correspond well with previously identified cytological locations of Hsf binding on salivary gland polytene chromosomes, thus providing direct target genes associated with the low-resolution cytological analysis. A comparison with studies using *S. cerevisiae *Hsf [27,28] suggest that a set of conserved genes are regulated by Hsf in both organisms. Overall, this study presents the strong potential of this approach for *in vivo *genome-wide mapping of transcription factor binding sites in higher eukaryotes using the whole organism.

## Results and discussion

### Immunopurification of Hsf-bound chromatin

To test the effectiveness of ChIP-array and assess the possibility of using genome tile arrays to map the *in vivo *location of transcription factor binding sites with intact whole organisms, we used the well characterized transcription factor Hsf, the mediator of the heat-shock response in *Drosophila*. Formaldehyde-crosslinked chromatin from *Drosophila *embryos was used as the input for immunopurifications with either anti-Hsf antisera or preimmune sera. After immunopurification and washing, the formaldehyde crosslinks were reversed by heating and the DNA purified. This DNA was initially analyzed for the enrichment of known Hsf targets by quantitative real-time PCR assays using a series of specific primers. We assayed the *Hsp26 *and *Hsp70A *genes with primers that amplify fragments spanning either the 5' HSE or a control 3' untranslated region (UTR) fragment of each gene. As shown in Table 1, the chromatin immunopurification shows both good enrichment and high specificity. With both *Hsp26 *and *Hsp70A *we observe over 100-fold enrichment of HSE fragments with anti-Hsf versus preimmune serum and a similar enrichment of HSE versus 3' ends with the anti-Hsf sera.

Because many of the published ChIP-array studies employ a ligation-mediated PCR step (LM-PCR) to amplify the enriched DNA, we assayed whether LM-PCR amplification of the DNA prepared from anti-Hsf immunopurifications maintained the enrichments we observe with unamplified material. We find that the enrichment of *Hsp *gene HSEs, as measured by quantitative PCR, is similar between amplified and unamplified material, demonstrating, at least with respect to the *Hsp *genes we examined, the validity of using LM-PCR amplification of ChIP-enriched DNA (data not shown). During the course of our experiments we tested embryos that had not been subjected to a heat shock but were processed in the same way as heat-shocked embryos. We found significant enrichment by quantitative real-time PCR (between 25- and 90-fold enrichment of HSEs in three independent experiments). Because considerable evidence indicates that Hsf is not specifically bound to HSEs in unstressed *Drosophila *cells [20], our observation suggests that the preparation of the embryos may have induced the stress response, possibly during the dechorionation step in bleach.

### Genome tile arrays

We assayed the effectiveness of using genome tile arrays to identify *in vivo *Hsf-binding sites. We constructed microarrays containing a total of 3,444 PCR products. These include 3,092 fragments representing 2.9 Mb of chromosome arm 2L, from *kuzbanian *to *cactus*, 96 fragments representing the regulatory regions for a set of early segmentation genes (*even-skipped*, *hairy*, *runt *and *Dichaete*) and a set of 95 products spanning fragments identified in a previous immunopurification experiment with anti-Ubx [2]. The fragments ranged in size from 282 to 1,380 bp with an average size of 930 bp (SD ± 53 bp). In addition to these we produced 162 fragments encompassing five different *Hsp *gene loci; regions of approximately 10 kb encompassing *Hsp68 *at 95D11, *Hsp83 *at 63B11, *Hsp60 *at 10A and *Hsp70A *at 87A2 along with a 22-kb region from 67B1 containing *Hsp67Bc*, *Hsp67Ba*, *CG32041*, *Hsp23*, *Hsp26 *and *Hsp27*. The *Hsp *gene regions were represented in two fragment sets: a set of 1-kb fragments overlapping by 500 bp and a set of 2-kb fragments overlapping by 1 kb. Finally, 480 elements were spotted with sheared *Drosophila *DNA to give a microarray containing 3,924 elements.

We prepared chromatin from heat-shocked embryos, performed immunopurification in parallel with anti-Hsf and preimmune sera and amplified the resulting purified DNA by LM-PCR. Each sample was independently labeled with a fluorescent dye, the labeled anti-Hsf and preimmune samples were mixed and then co-hybridized to the tiling path microarrays. We performed dye-swap experiments to assess any bias in the incorporation of the fluorescent dyes. We used three independent biological replicates and for each preparation performed technical replicates, in total carrying out 11 separate hybridizations (see Additional data file 1 for the full data).

**Figure 1:**
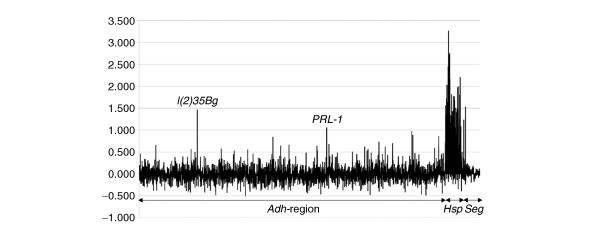
Distribution of fragment enrichment with anti-Hsf immunopurified chromatin on the genomic tiling array. The *y*-axis plots the asinh transformation (approximately equivalent to the log_2 _scale) of the ratio of anti-Hsf versus preimmune sera. The *x*-axis represents each of the 3,444 PCR products, the *Adh *region, *Hsp *gene and segmentation gene (*Seg*) sequences are indicated below the *x*-axis. Strong enrichment of fragments from the *Hsp *genes is indicated by their high ratio. The signals from *l(2)35Bg *and *PRL-1 *in the *Adh *region are indicated.

After normalization, we calculated the ratio of anti-Hsf signal to the preimmune signal. Ratios for each technical replicate were averaged and the average ratios used to calculate a probability score for each spot using Cyber-T [29]. The 480 sheared genomic DNA fragments were distributed evenly across the slide and allowed us to evaluate the consistency of input DNA samples; these had an average asinh ratio of -0.13 ± 0.09 (standard error = 0.004, variance = 0.009) indicating no significant overall difference between the samples. Of the 3,444 elements containing PCR-amplified fragments of *Drosophila *DNA, 59 showed a greater than 1.6-fold enrichment (up to 10-fold enrichment) with the DNA purified with anti-Hsf sera at *p*-values better than 10^-3^. Of these elements, 53 (88%) correspond to fragments from *Hsp *gene loci, five from the *Adh *region and one from the putative Ubx target set. Plotting the average ratio for each array element with respect to the order of the fragments on the genome (Figure 1), we observe a striking distribution of signal; the fragments derived from the *Adh *region and the segmentation genes show little signal above asinh ratios of 0.5, with only four fragments showing more than twofold enrichment. In contrast, many fragments from the *Hsp *gene regions show substantial enrichment. Of the 162 fragments from the *Hsp *gene loci, 46 show greater than twofold enrichment with the anti-Hsf sample. The results are highly reproducible; comparing the ratios obtained with the 162 *Hsp *fragments from each of the replicate slides, the correlation between any two slides ranged from 0.7 to 0.98, with an average correlation of 0.84.

**Figure 2:**
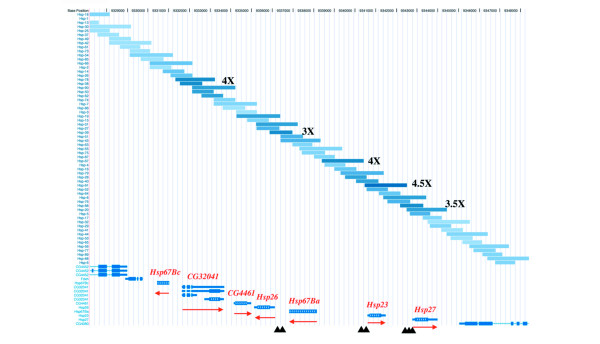
Graphical representation derived with the University of California at Santa Cruz (UCSC) genome browser of fragment enrichments in the 67B region containing eight putative *Hsp *genes (*CG32041 *encodes a dicistronic transcript). The blue fragments represent the 1-kb and 2-kb tiling fragments with the intensity of the blue color reflecting the degree of enrichment (asinh ratio); selected regions have been labeled with fold enrichments. The direction of transcription for each of the *Hsp *genes is indicated by the red arrow. The black triangles at the bottom indicate the locations of known HSEs.

The distribution of the signals across the *Hsp *genes shows excellent agreement with the known location of HSEs at the 5' end of the transcription units and, in addition, show a monotonic signal distribution centered on the fragments containing HSEs. This is best exemplified by the 20-kb region, which encompasses the eight known or putative *Hsp *genes in the 67B region (*Hsp67Bc*, the bicistronic *CG32041*, *CG4461*, *Hsp26*, *Hsp67Ba*, *Hsp23 *and *Hsp27*) where we observe strong enrichment of fragments close to the 5' ends of heat-inducible genes and negligible signals in between (Figure 2). Five clear peaks of fragment enrichment are observed and there is good overlap with the known locations of Hsf-binding sites [30]. A major peak 5' to *Hsp26 *encompasses the characterized Hsf-binding sites at -349 and -56. Three further peaks cover the regions of the 5' ends of *Hsp67Ba*, *Hsp23 *and *Hsp27*, including the known HSEs upstream of *Hsp23 *(-391 and -119) and *Hsp27 *(-366, -328 and -270). Finally, a fifth peak overlaps the 5' ends of the divergent transcription units of *Hsp67Bc *and *CG32041*, the latter being a dicistronic gene encoding *Hsp22 *and *Hsp67Bb*. There appears to be no substantial enrichment covering the 5' end of the Hsp20-like *CG4461*; however, it is not known if this gene is Hsf-inducible. Thus seven out of the eight *Hsp *genes in the region have 5' regions enriched by our assay. Fragments including known HSEs show the highest enrichments (more than 3.5-fold), whereas nearby fragments show no significant signal over the background. This region demonstrates the potential for high-resolution mapping of *in vivo *DNA binding and suggests that even gene-dense regions can be accurately mapped using the ChIP-array technique with 1-kb tiling paths.

**Figure 3:**
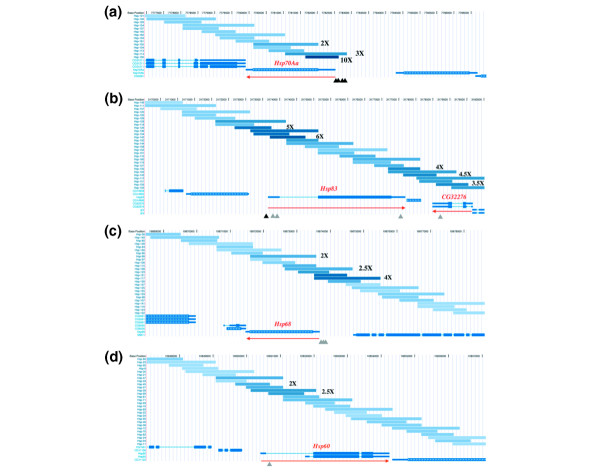
Graphical representation of fragment enrichments for four *Hsp *gene regions derived with the UCSC genome browser. Details as for Figure 2; gray triangles represent predicted Hsf-binding sites. See text for details. **(a) ***Hsp70A*; **(b) ***Hsp83*, note the enrichment both 5' and 3' to the gene; **(c) ***Hsp68*, enriched fragments 5' to the gene contain predicted Hsf-binding sites; **(d) ***Hsp60*, the enriched fragments within the intron contain predicted Hsf sites.

The other *Hsp *gene loci show similar distributions of fragment enrichment (Figure 3). With Hsp70, three fragments show greater than twofold enrichment with the two fragments (Hsp-130 and Hsp-114) encompassing the known *Hsp70A *regulatory elements, several HSEs between -252 and -46 bp [30], showing the greatest enrichment (Figure 3a). In the case of *Hsp83 *we see a different organization, and Hsf binding is not restricted to the immediate 5' region (Figure 3b). We observe two strong peaks of signal enrichment. One centers on the area immediately 5' to the start of Hsp83 expression where HSEs have been mapped between -88 and -49 [30]. However, the ChIP also reveals a second peak at the 3' of *Hsp83 *extending to cover *CG14966 *(a gene of unknown function) and 3' to *CG32276*, a predicted chaperone. This additional signal contains matches with an Hsf consensus binding sequence, suggesting that it represents a *bona fide *Hsf-binding site. It has previously been noted that *Hsp83 *stands out from other Hsp genes in the dynamics of its response to heat shock [24] and this may be linked to the distinct arrangement of Hsf-binding sites we find.

With *Hsp68 *we find that two overlapping fragments show greater than fourfold enrichment (Hsp-117 and Hsp-131) and these correspond to the region immediately 5' to the start of *Hsp68 *transcription; the fragments flanking these are also detected with lower ratios (Figure 3c). Although there are no reports of mapping Hsf-binding sites in the *Hsp68 *region, we find three perfect matches to a consensus Hsf-binding site 160 bp upstream of the mRNA start site, consistent with the fragment enrichment we observe. Finally, with the *Hsp60 *gene we observe moderate but clear enrichment with fragments encompassing the first intron of the gene, and also find a match to a consensus HSE sequence in this region (Figure 3d, see below). *Hsp60 *is reported not to be induced by heat shock in *Drosophila *and previous studies have failed to find HSE sequences 5' to the start of *Hsp60 *transcription [31]. In mammals and yeast, however, *Hsp60 *homologs are heat inducible [32,33] and our data indicate conservation of Hsf binding.

As well as the *Hsp *genes, we observe a greater than twofold enrichment with two fragments in the *Adh *region (Figure 1). One fragment maps between the divergently transcribed genes *l(2)35Bg *and *Su(H) *suggesting that either of these genes could be regulated by Hsf. Supporting this suggestion, we find that *l(2)35Bg *gives a strong positive signal when independent anti-Hsf immunopurifications are used to interrogate the cDNA arrays described below. In the second case, we observe a twofold enrichment of a fragment overlapping the 5' end of the longest transcript from the *PRL-1 *gene and we also observe a weak enrichment (1.2-fold) of a fragment overlapping a second transcription start-site 5 kb downstream (data not shown). Interestingly, the *PRL-1 *gene was identified by Sun *et al. *[15] as a candidate GAGA-factor (Gaf)-regulated gene in their DamID analysis of the *Adh *region. In some cases, most notably *Hsp70A *and *Hsp26*, Hsf- and Gaf-binding sites are located in close proximity and are both involved in transcriptional regulation of *Hsp *genes [34].

In addition to the fragments showing greater than twofold enrichment, we find a further eight fragments showing greater than 1.5-fold enrichment with the anti-Hsf immunopurification. Some of these may represent weak Hsf-binding sites. For two of these regions (*CG4500 *and *CG3793*) we detect enrichment in the experiments with the cDNA arrays described below, suggesting that they may represent *bona fide *Hsf-binding sites in the genome.

To try and assess the validity of the fragments identified on the array and relate the degree of enrichment with the presence of HSE, we used the informatics tool MEME [35] to examine the enriched fragments for the presence of consensus Hsf-binding sites. As noted above, we find predicted Hsf-binding sequences in the regions enriched upstream of *Hsp68*, downstream of *Hsp83 *and in the intron of *Hsp60*. We also find potential Hsf-binding sequences within the fragments enriched from the *Adh *-region, indicating that enrichment on the tiling arrays corresponds to the location of some Hsf-binding sites. Taken together, the experiments and analysis described above demonstrate that chromatin immunopurification used in tandem with tiling DNA microarrays can successfully identify genuine *in vivo *transcription factor binding sites at the level of the whole organism. Our mapping suggests locations for new HSE elements regulating *Hsp83*, *Hsp68 *and *Hsp60*.

### Genome-wide search for HSF target genes

Since much previous work, along with the observations presented above, indicates that the binding sites for Hsf tend to be located close to the transcriptional start of responsive genes [24], we reasoned that we could identify new genes with Hsf-binding sites by performing a ChIP-array analysis using arrays containing cDNA clones. To this end we utilized a microarray containing 5,372 full-length cDNA clones representing 5,073 genes, prepared from the *Drosophila *Gene Collection V1.0 [36]. We performed immunopurifications using anti-Hsf and preimmune sera on chromatin isolated from three independent biological preparations. In addition, to assess reproducibility, we performed independent immunopurification reactions with two of the chromatin preparations. With chromatin A we performed four separate immunopurifications (1-4); the first two of these were technically replicated as well as dye-swapped and the second two were dye-swapped only. From chromatin B we performed two independent immunopurifications and each of these were dye-swapped. With chromatin C we performed a single immunopurification and dye-swap (full data in Additional data file 2). In total we performed 18 hybridizations to the cDNA arrays. The average correlation between each technical replicate was very high (> 0.85) and after generating an average ratio for each technical replicate we used the CyberT algorithm to generate *p*-values from the average ratios for each independent immunopurification.

**Figure 4:**
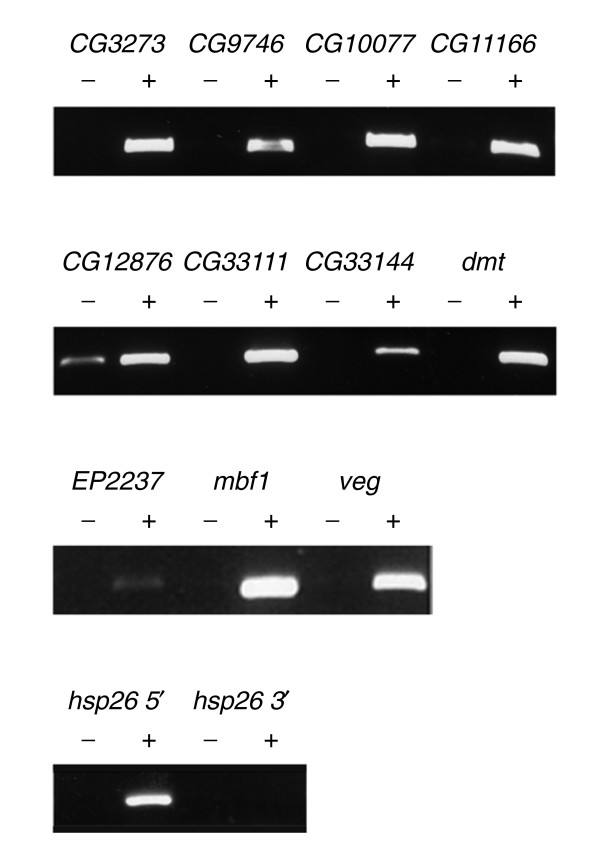
PCR validation of selected positives from the cDNA arrays. Agarose gels showing the products generated by specific PCRs for each of the indicated genes using preimmune purified (-) or anti-Hsf purified (+) chromatin as an input.

We identified 188 genes that showed greater than 1.6-fold enrichment. While we recognize that defining an enrichment cutoff in the absence of other data is somewhat arbitrary, we selected a 1.6-fold value based on the enrichments observed on the genome tiling arrays with known Hsf-binding sites. We note however that this criterion may underestimate the Hsf-binding targets as the cDNA array elements will only detect binding sites close to the 5' end of the cDNA. Genes that bind Hsf at more distant sites will be expected to generate weaker signals on the array that will escape detection owing to noise issues with low signals. To validate the Hsf targets we selected 11 genes distributed across the ranking from 1 to 188, and tested for enrichment of the 5' genomic DNA upstream of each gene in a standard ChIP assay along with 5' and 3' end of *hsp26 *as a control. As shown in Figure 4, all 11 genes tested showed clear enrichment when DNA derived from anti-Hsf sera and preimmune sera are compared. Thus the microarray assay is in excellent agreement with standard PCR assays and suggests that, at least with the enrichments we observe, the ChIP-array data is highly reliable. Of the 188 genes with the selected 1.6-fold enrichment, 141 were enriched with *p*-values of 9 × 10^-3 ^or better. Enrichments as high as eightfold were reproducibly observed and, reassuringly, enriched genes include a number of *Hsp *genes along with other predicted chaperone-encoding genes such as *DnaJ-1*, *CG32041 *and *CG32649 *(Table 2). Using the stringent *p*-value cutoff, our analysis indicates that approximately 3% of the genes in the *Drosophila *genome (around 400) may be direct targets of Hsf, a figure that is in remarkable agreement with a recent analysis of Hsf binding in *S. cerevisiae *[28].

In general, the agreement between the independent immunopurifications and the different chromatin samples was very good, however we noticed that each immunopurification identified a set of genes that showed no significant enrichment in other samples. These 'IP-specific' signals were consistent within the technical replicates and showed high enrichments (up to sevenfold). They did not, however, correlate with a particular chromatin preparation, since there was no similarity between the different immunopurifications performed from the same chromatin. We assume that these artifacts reflect the inherent noisiness of the system and emphasize the need to perform replicate immunopurifications from particular biological samples in order to identify consistently positive signals.

**Figure 5:**
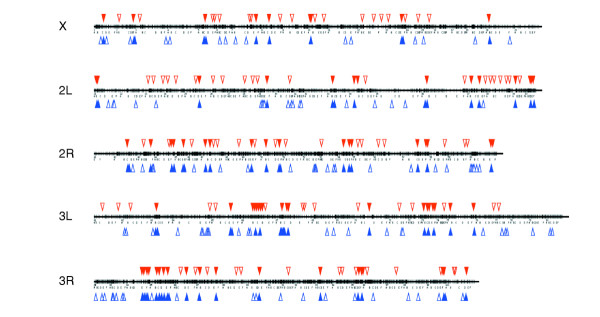
Representation of the predicted cytological location of the top 188 Hsf-binding genes. Those identified with our cDNA array are indicated by blue triangles and the mapping of Hsf sites on polytene chromosomes reported by Westwood *et. al. *[22] is shown by red triangles. Filled triangles represent matches between the two studies and open triangles represent unmatched mapping.

We determined the predicted cytological location of the all 188 top Hsf target genes and compared this list to the cytological mapping of Hsf-binding sites on polytene chromosomes, which is, of course, quite low resolution [22]. Of these genes, 82 are predicted to map to the same cytological band as an Hsf site (50%) and a further 40 are predicted to map within a lettered division of a site mapped by Westwood *et al. *[22] (Figure 5). Thus from the 164 cytological sites reported to bind Hsf immediately after heat shock, we have identified 122 (75%) candidate genes as Hsf targets in these locations with our survey of approximately 40% of the predicted genes in the genome.

We examined the expression of the cDNAs on the array by hybridizing with labeled cDNA prepared from heat-shocked embryos compared to unshocked controls; 16 of the top 188 genes showed induction greater than 1.7-fold (Table 2) with known heat-shock response genes being robustly induced; for example, over 30-fold increases in Hsp26 and Hsp27 expression. A further two genes are repressed more than twofold. We examined the only other reported *Drosophila *array data, obtained from custom oligonucleotide arrays hybridized with RNA derived from heat-shocked and non-heat-shocked embryos [37]. Of the genes represented on the custom array, 21 are found in our top 188 Hsf-binding genes; of these, seven genes (*Hsp26*, *27 *and *23*, *DnaJ-1*, *Hsc70-5*, *CG3488 *and *Cct-gamma*) show induction and one (cyclophilin 1; *Cyp1*) is repressed, according to the quality criteria used by the authors. In general the data are in reasonable agreement; however, we find no evidence with our cDNA array for induction of *Cct-gamma *and *CG3488 *or repression of *Cyp1*. These discrepancies may reflect strain differences, platform-specific signals or experimental noise. We conclude that only a minority of the Hsf targets that we have identified show clear evidence of direct induction or repression using our heat-shock regimes and sampling times.

In a recent Hsf1 ChIP study of mammalian cell lines, approximately 50% of the 94 identified Hsf1-bound promoters did not directly produce heat-induced transcripts [38], leading to the interpretation that Hsf binding alone may not confer heat-inducibility. Indeed it is clear that even in the well characterized Hsp gene regulatory regions, Hsf collaborates with other transcription factors [39]. In contrast, Hahn *et al. *[28] were able to use the extensive expression data available in yeast to determine what fraction of the 165 Hsf targets they identified by ChIP showed evidence of induction by heat shock. Only 7% of the putative Hsf targets did not show evidence of heat-shock induction. In multicellular eukaryotes, with the possibilities of considerable developmental and tissue-specific effects on gene expression, more extensive expression analyses will be required to enable us to address the question of how many of the Hsf target sites are associated with Hsf-mediated regulation of expression.

**Figure 6:**
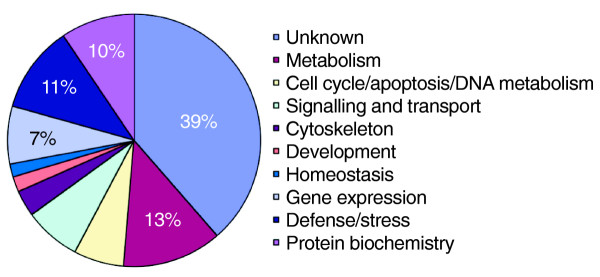
Gene ontology classification of the top 188 genes identified from the cDNA array. Percentage representations are given for the prominent categories.

We used the Gene Ontology (GO) annotation to classify the gene products represented by the 188 Hsf-bound genes (Figure 6). As would be predicted, proteins annotated with chaperone or chaperone ATPase activity are well represented; we find 17 chaperones among the Hsf target genes. Using GeneMerge to assess enrichment of GO terms in the Hsf targets compared to all of the genes on the array, we find highly significant enrichment of genes with chaperone or heat-shock protein activity (*p *< 8 × 10^-6^) functional annotation. In terms of biological processes, response to heat or temperature are over-represented (*p *< 2 × 10^-4^) (Figure 5). In addition, we find 18 genes involved in basic metabolism, in protein modification or degradation, 12 genes associated with the cell cycle or programmed cell death and, interestingly, 14 genes associated with gene expression. Of this latter class, eight are documented as showing changes in expression in response to dietary changes or oxidative stress [40,41] and this suggests a link between downstream components of different stress responses. Of particular interest are four genes (*Taf7*, *CG33097*, *TfIIEα *and *Trap36*) that encode core components of the RNA polymerase II transcription machinery. *Trap36 *is a component of the Mediator complex, which has been shown to play a vital role in transcriptional induction by Hsf at the *Hsp70A *promoter [42]. These data suggest that part of Hsf function may be to regulate components of the core transcriptional machinery necessary for the stress response in order to modulate or temporally control the response.

As noted above, in some cases heat-shock responsive genes may be regulated by both Hsf and Gaf. A recent study identified potential binding targets of Gaf by the Dam-ID technique using cDNA arrays very similar to those used here [16]. We therefore examined the overlap between the sets of genes binding both factors. Of the 188 Hsf-binding genes, 39 were identified as being potential Gaf targets (>1.4-fold enrichment *p *< 10^-3^, Table 2). Of these we find, as expected, the chaperones *Hsp22*, *Hsp23*, *Hsp26*, *Hsp27 *and *DnaJ-1*. There is no obvious correlation between high expression and binding of both Hsf and Gaf. Although the highly expressed chaperones discussed above appear to be targets of both Hsf and Gaf, four other chaperones (*CG7945*, *Hsc70Cb*, *Hsc70-5 *and *CG32649*), which are induced by heat shock, bind only Hsf and not Gaf. Of interest in the set of genes bound by both factors is the TGFβ receptor *thick veins*, as well as three annotated transcriptional regulators (*Taf7*, *CG6792 *and *GATAd*). This suggests that a complex secondary response to stress may involve co-regulation of key transcriptional and signaling regulators by both Hsf and Gaf.

**Figure 7:**
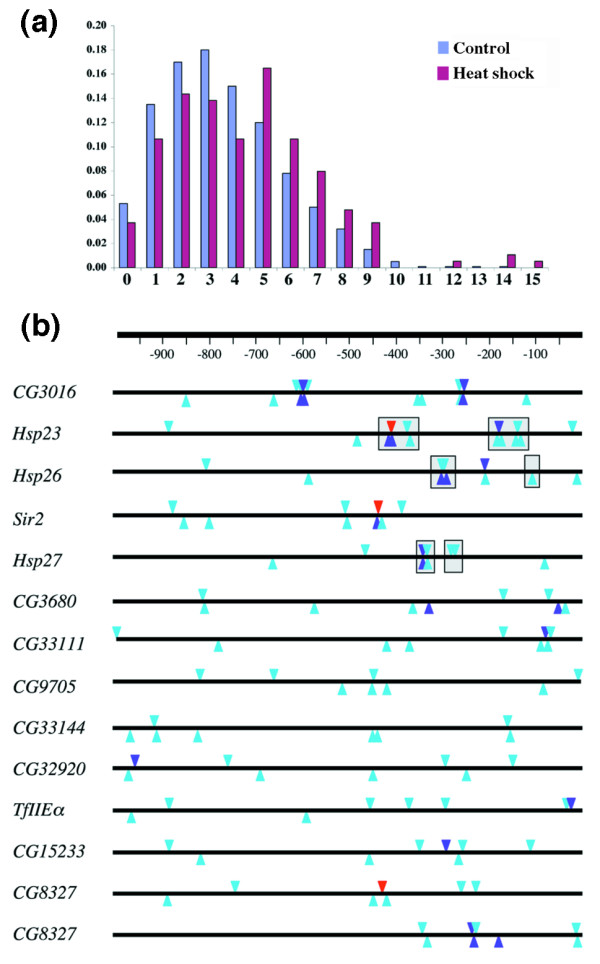
Predicted Hsf-binding sequences in the 1-kb region upstream of Hsf-binding genes. **(a) **Plot of the distribution of the number of predicted sites as a proportion of the population of anti-Hsf-enriched (Heat shock) or non-enriched (Control). **(b) **The relative position of predicted Hsf sites for each of the genes containing eight or more sites. The annotated gene start is on the right. Red triangles, perfect match; purple, one mismatch; light blue, two mismatches. Gray boxes represent the known HSEs upstream of *Hsp23*, *Hsp26 *and *Hsp27*.

We next sought to determine whether the sequences upstream of the top Hsf-binding genes were enriched for potential Hsf-binding sites. We used standard pattern matching software to look for matches to a consensus Hsf-binding site TTCnnGAAnnTTC [43] in the 1 kb immediately upstream of the top-ranked 188 Hsf-binding genes. As a control we examined the 1-kb regions upstream of the 5,000 genes on the array that showed no enrichment with Hsf. Plotting the number of predicted Hsf sites against the number of genes shows that for both the anti-Hsf enriched and the non-enriched sequences there is a broadly similar distribution for upstream regions containing five or fewer matches to the consensus (Figure 7a). However, in the case of the anti-Hsf enriched fragments we find an over-representation of upstream regions that contain six or more consensus Hsf sites. These include, as expected, the known heat-shock genes (*Hsp23, Hsp26 *and *Hsp27*) but also genes for transcription factors (*TfIIEα *and *CG6197*) and genes of unknown function. In most of these cases we find that predicted Hsf sites are clustered and preferentially located within 500 bp upstream of the transcription start (Figure 7b). This supports the view that the sites we have identified represent genuine HSEs. We also observe that the number of predicted Hsf sites is not related to the fold enrichment we observe on the microarrays, suggesting either that fragment enrichment is not an accurate measure of Hsf 'binding affinity' or that simple binding site prediction is not a reliable way of identifying genuine HSEs.

### Comparative analysis

Two genome-wide studies in the budding yeast *S. cerevisiae *have mapped the location of *HSF1 *by ChIP-array. In one case, Hsf binding was determined using unstressed cultures and over 100 potential targets identified with significant *p*-values according to the error model used by the authors [27]. In a second study, using both unstressed and heat shocked cells, 165 genes were identified with Hsf1-binding sites that showed enrichment above a threshold set by consideration of heat-inducible expression [28]. We compared our data from the *Drosophila *cDNA array with this yeast data to look for similarities in the sets of genes potentially regulated by Hsf in both organisms. Taking the protein sequences of the top hits from the cDNA array, we looked for yeast genes encoding proteins with BLAST matches better than 1e-10 and identified 83 genes. We then examined their enrichment in the yeast Hsf-binding datasets. These data are summarized in Table 3. Using the cutoff criteria employed by Hahn *et al. *[28] we find 11 yeast genes that are predicted to bind Hsf and another gene (*CG4800*) just below their threshold. A further 11 genes, identified in the Lee *et al. *data [27] with *p*-values better than 1 × 10^-2 ^are also conserved. This set of 23 Hsf target genes conserved between fly and yeast not only includes characterized heat-shock response genes (*DnaJ-1 *and *Hsc70-5*) but also seven other putative stress-response genes (including *Hsp60*), 12 genes with other functions and two genes of unknown function. This clearly represents a minimal set as it is limited by identification of homologous genes and by cross-comparability of the datasets. For example, the small Hsp20-like chaperones are not conserved in sequence between fly and yeast, although proteins with similar functions are clearly bound by Hsf in both organisms. Since we have only surveyed approximately 40% of the *Drosophila *genome, it suggests a minimal core of over 50 genes as a conserved set of eukaryotic Hsf targets.

Along with chaperone-encoding genes, we find other genes whose products are suggested to be implicated in stress responses; *CG4800*, a putative microtubule-binding protein associated with the defense response and *cyclophilin1. CG1416 *encodes a protein with a possible Hsp90 interaction domain, which, according to the data from a *Drosophila *gene-expression time course [44], is coexpressed with two genes: *foraging*, encoding a cyclic-nucleotide dependent protein kinase [45], and *effete*, a predicted ubiquitin-conjugating enzyme [46]. We find that both these genes are bound by Hsf in *Drosophila*, albeit with lower enrichments than *CG1416 *(1.5- and 1.6-fold) and homologous genes are also bound in yeast (the protein kinase *TPK2*, with a modest 2.8-fold enrichment and *UBC4*, a ubiquitin-conjugating enzyme with a highly significant enrichment (*p *= 1.1e-4). This suggests the possibility that these proteins may interact in a common stress-response pathway.

Among the remaining genes, *l(2)35Bg *represents a highly conserved protein found throughout eukaryotes. While the function of this protein is unknown, mutations in yeast and *Drosophila *are lethal, in the latter case lethal in embryos. Our findings suggest that *l(2)35Bg *encodes a conserved factor involved in the stress response.

Of particular interest among the conserved Hsf targets is the helix-turn-helix containing transcription coactivator multiprotein bridging factor 1 (Mbf-1). This protein has been shown to mediate the interaction between nuclear hormone receptors and TATA-binding protein (TBP) in both *Drosophila *and mammalian systems [47,48] and plays a similar role in yeast, where it is involved in mediating the interaction between TBP and the leucine-zipper transcription factor GCN4. Null mutants in yeast are viable but sensitive to amino-acid deprivation [49]. In *Drosophila *the gene is strongly induced by oxidative stress (paraquat treatment [40]), moderately induced by heat shock (this paper) and repressed under starvation conditions [41]. Recent reports suggest that *mbf-*1 mutants are also viable in *Drosophila *but are sensitive to oxidative stress [50]. This report further suggests that Mbf-1 interacts with the c-Jun/c-Fos AP-1 dimer to mediate AP-1 stress-response activity. These observations suggest that there may be an underlying link between different types of stress response (heat, oxidation and nutritional) and that Mbf-1 may be intimately involved in the transcriptional response to environmental conditions, playing a vital role in coordinating the interaction of different stress-response transcription factors with the core RNA polymerase II complex.

## Conclusions

We have used chromatin immunopurification in conjunction with genome tiling and cDNA microarrays to map the *in vivo *binding sites of the heat-shock factor Hsf. Our results demonstrate the potential for mapping *bona fide *transcription factor binding sites at a genome-wide scale in complex multicellular eukaryotes. We find that the technique is highly reproducible and, with appropriate experimental replication, can identify binding regions with high fidelity. We further demonstrate a core set of Hsf targets conserved between fly and yeast that may represent a evolutionarily conserved regulatory network. The response of an organism or cell to stress is highly complex and necessitates direct control of physiological processes as well as modulation of gene transcription. The set of Hsf targets we identify includes many metabolic enzymes, which may be candidates for control points directly controlling metabolic and physiological processes in times of stress. The finding that several genes encoding transcriptional regulators are bound by Hsf, in particular components of the core RNA polymerase complex, suggests that one of the roles of Hsf may be in initiating or establishing a transcriptional state necessary for recovery from heat stress as well as its more traditional role in activating immediate stress-response genes. In both flies and mammalian systems Hsf target genes are not all immediately transcriptionally induced, suggesting that the heat response may be more complex than simply activating chaperones. In addition, the observation that Hsf may be regulating genes implicated in other stress responses suggests that responses to different stresses may involve underlying similarities. The extension of these studies to full genome coverage in *Drosophila *as well as other tractable model systems such as *Caenorhabditis elegans*, offers the prospect of understanding the regulatory response underpinning a fundamental cellular process.

## Materials and methods

### Anti-Hsf antiserum

We generated specific rabbit polyclonal antisera against a bacterially expressed *Drosophila *Hsf (CG5748). Briefly, we used a construct (MBP-dHsf [25], kindly provided by J. Lis, Cornell University) to produce a fusion protein containing the first 691 amino acids of Hsf fused to maltose-binding protein. After excision from an SDS-polyacrylamide gel, the gel slice containing the fusion protein was used as antigen in rabbits (approx 100 μg per rabbit per immunization) to produce a high titer antiserum (Eurogentec, Seraing, Belgium). The specificity of the antiserum for Hsf was confirmed by western blots of *Drosophila *nuclear extracts, where a band of approximately 110 kDa is recognized, as expected for *Drosophila *Hsf [22]. In addition, immunolabeling of *Drosophila *embryos with the anti-Hsf antiserum gives the expected ubiquitous nuclear staining, which is absent from embryos labeled with the preimmune serum and from *hsf*-null embryos labeled with the anti-Hsf antiserum (two Hsf null conditions were tested; *hsf*^1^and *Df(2R)ED3610 *homozygotes).

### Chromatin immunopurification from *Drosophila *embryos

Embryos (1-2 g) were collected over a 16 h period and then heat shocked for 15 min at 37°C. After the embryos were dechorionated in weak bleach (5% w/w available chlorine) for 3 min they were washed in H_2_O and then in PBS/0.01% Triton (PBST). The embryos were then centrifuged (1 min at 500 *g*) and resuspended in 10 ml crosslinking solution (50 mM HEPES pH 8.0, 1 mM EDTA.Na_2, _0.5 mM EGTA, 100 mM NaCl) containing formaldehyde (1.95%) and 30 ml n-heptane. This was incubated at room temperature with vigorous shaking for 15 min. The fixed embryos were centrifuged (1 min at 500 *g*), resuspended in PBST-glycine (PBST, 125 mM glycine) and allowed to sediment. After the embryos were washed with ice-cold PBST, they were again allowed to sediment. The supernatant was removed and the embryos were resuspended in 15 ml ice-cold PBST containing protease inhibitors. After douncing using a Wheaton Dounce Tissue Grinder (pestle B), and centrifugation at 400 *g *at 4°C for 1 min, the supernatant was removed and centrifuged at 1,100 *g *for 10 min at 4°C. The pellet was resuspended in 15 ml ice-cold cell lysis buffer (5 mM PIPES pH 8, 85 mM KCl, 0.5% Nonidet P-40) containing protease inhibitors and dounced using a Wheaton Dounce Tissue Grinder (pestle A). Then the extract was centrifuged at 2,000 *g *for 4 min at 4°C and the pelleted nuclei were resuspended in 3 ml ice-cold nuclear lysis buffer (50 mM Tris.HCl pH 8.1, 10 mM EDTA.Na_2_, 1% SDS) including protease inhibitors. After 20 min at 4°C, 0.3 g acid-washed glass beads (Sigma, 212-300 μm diameter) were added and the extract was sonicated using a heat systems ultrasonic liquid processor XL sonicator with a microtip attached. The extract was exposed to a 1 × 30 sec burst at level 3, and 5 × 30 sec bursts at level 4 with 90 sec resting on ice between bursts. Fragment sizes between 0.5 and 1 kb were produced. The chromatin extract was clarified by centrifugation at 14,000 rpm for 10 min at 4°C, flash frozen in liquid nitrogen and stored at -80°C.

Chromatin immunopurification was performed according to the method of Oberley *et al., *[51]. Briefly, the chromatin solution was diluted with IP dilution buffer (16.7 mM Tris.HCl pH 8, 167 mM NaCl, 1.2 mM EDTA.Na_2_, 1.1% Triton X-100, 0.01% SDS) and precleared with fixed and killed *Staphylococcus aureus *Protein A-positive strain cells (SAC) for 15 min. The precleared diluted chromatin sample was incubated with 1 μl of either preimmune serum or anti-Hsf serum overnight at 4°C. To capture the antibody-chromatin complexes, SAC were added and the samples were incubated for 15 min at room temperature. The SAC were washed twice in IP dialysis buffer (50 mM Tris.HCl pH 8, 2 mM EDTA.Na_2_, 0.2% sarkosyl) and four times in IP wash buffer (100 mM Tris.HCl pH 9, 500 mM LiCl, 1% deoxycholic acid, 1% Nonidet P-40). The immunopurified material was eluted from the SAC by vigorously vortexing for 15 min in elution buffer (50 mM NaHCO_3_, 1% SDS). RNase A was then added (33.3 μg/ml) and NaCl to 0.3 M. To reverse the crosslinks the material was incubated for 5 h at 67°C and then precipitated with ethanol. Proteinase K (0.6 units/ml) was added and the samples were incubated at 45°C for 2 h and purified with one extraction with phenol/chloroform/isoamyl alcohol followed by a chloroform extraction. After precipitation with ethanol in the presence of glycogen the DNA was resuspended in TE buffer.

### Quantitive real-time PCR

Quantitive real-time PCR experiments were performed with a Corbett Research RotorGene utilizing SYBR Green fluorescence. Reactions were carried out in 15 μl using SYBR Green PCR master mix according to the manufacturer's protocol (Qiagen) with 2.4 μl DNA. Cycling was for 15 min at 95°C, followed by 40 cycles of 94°C, 60 sec; 60°C, 30 sec and 72°C, 60 sec. The primer pairs to amplify heat-shock element and 3' ends of the genes for heat-shock proteins 26 (3' UTR) and 70 (5' HSE and 3' UTR) were as described in Andrulis *et al. *[25] except the primer pair (5'-GCTGTTTCTTTTGCGCTCTT and 5'-TTGTTTGACTTGTAAGCAAAGGTT) for the heat-shock element of heat-shock protein 26 (5' HSE). Serial dilutions of genomic DNA (100-0.3125 pg/μl) were used to produce a calibration curve. A no-template control was also used. All samples, controls and standards were performed in triplicate.

### Standard PCR

Positives from the cDNA microarray were validated in standard PCR assays as follows: To 3 μl of immunopurified DNA, 1 μl of 100 pmol/μl primers, 1.5 μl 10x buffer IV (Abgene), 1.5 μl 10 mM dNTPs, 1.2 μl 25 mM MgCl_2_, 1 μl Thermo-Start Taq DNA polymerase (Abgene, 5 units/μl) and H_2_O to 15 μl were added. PCR reactions were carried out for 5 min at 95°C, 35 cycles of 1 min at 95°C, 1 min at 57°C and 1 min at 72°C, followed by 10 min at 72°C. The primers used are listed in Table 4.

### Sample labeling

Concentrations of anti-Hsf and preimmune IP DNA samples were determined using a NanoDrop spectrophotometer (Nanodrop Technologies). Fifty nanograms of each IP sample was incubated with 1 unit T4 DNA polymerase (Promega) in a total volume of 50 μl manufacturer's buffer for 5 min at 37°C. The reaction was stopped by adding 2 μl of 0.5 M EDTA and the DNA purified with MinElute PCR purification columns (Qiagen). Ten nanograms of purified DNA was combined with 1 μM of annealed linkers (Linker 1, 5'AGAAGCTTGAATTCGAGCAGTCAG3': Linker 2, 5'CTGCTCGAATTCAAGCTTCT 3') and incubated overnight at 4°C with 1 unit of DNA ligase (Invitrogen) in standard ligase buffer.

PCR amplification was carried out directly without further DNA purification in a reaction volume of 100 μl containing 0.2 mM dNTPs, 15 mM MgCl_2_, 5 U Thermo-Start DNA polymerase (Abgene) and 100 pg of linker 2 using the following conditions: 1 cycle of 55°C 2 min, 72°C 5 min, 94°C 5 min; 24 cycles of 94°C 1 min, 55°C 1 min, 72°C 1 min; 1 cycle of 72°C 5 min, 4°C hold. PCR products were purified with MinElute columns (Qiagen).

### Labeling

Purified PCR products were labeled with a Bioprime random priming labeling system with 0.1 mM each dATP, dGTP and dTTP, 0.04 mM dCTP and 0.06 mM Cy3 or Cy5-conjugated dCTP (Amersham Biosciences) at 37°C for 2 h. 5% of the reaction was checked by agarose gel electrophoresis for an expected smear of product from 200-600 bp and the remainder purified with Sephadex G50 minicolumns.

### Tiling path microarrays

A total of 3,091 fragments of 1 kb average length were amplified with primers designed across the Adh region by PCR (coordinates chr2L:13488459-16409825; these primers were generously donated by P. Spellman and G. Rubin, University of California at Berkeley). All sequence coordinates are from release 3.1 of the *Drosophila *genome sequence [52]. The primer design was generated against release 1 of the genome sequence and we have remapped the fragments onto release 3.1 of the sequence using the UCSC genome browser [53,54]. In addition we synthesized three sets of primers to amplify the following loci. The first was a set of 1-kb and 2-kb overlapping fragments covering several *Hsp *gene loci: *Hsp70A *(chr3R:7,776,000-7,830,000), *Hsp83 *(chr3L:3,170,043-3,180,013), *Hsp67Ba-Hsp27 *(chr3L:9,326,084-9,348,399), *Hsp68 *(chr3R:19,868,471-19,878,621) and *Hsp60 *(chrX:10,847,030-10,857,109). The second was a set of 1-kb fragments covering a set of segmentation genes: *Eve *(chr2R:5,035,032-5,051,166), *Dichaete *(chr3L:14,096,994-14,125,069), *Hairy *(chr3L:8,619,968-8,637,146) and *Runt *(chrX:20,349,976-20,380,172). The third was a set of 81 1-kb fragments corresponding to regions previously identified by ChIP with an anti-Ubx antibody [2]. All primer sequences are given in Additional data file 4.

All PCR amplifications were performed in 96-well plate format and each product was assayed by agarose gel electrophoresis. In total, 3,444 fragments were amplified and spotted, along with 480 samples of sonicated *Drosophila *genomic DNA (250 ng/μl), onto FMB-cDNA glass microarray slides with a Biorobotics Microgrid II arrayer. cDNA arrays were constructed from PCR amplified inserts from the *Drosophila *Gene Collection V.1 [36] and are described at the FlyChip website [55].

Slides were treated as described on the FlyChip website [55]. Slide hybridization and washing was carried out with a Genomic Solutions GenTAC hybridization station. Slides were scanned with an Applied Precision ArrayWoRx CCD scanner and the data processed using a custom implementation of the VSN normalization method of Huber *et al. *[56]. VSN performs an asinh transform with the microarray ratio data rather than the more traditional log_2 _transformation; in most cases the two are equivalent. The CyberT framework and website was used to assess the significance of the microarray results [29,57]. Yeast data were obtained from Lee *et al. *[27] and Hahn *et al. *[28] and the mammalian data from Trinklein *et al. *[38].

Expression analysis comparing RNA from heat-shock treated embryos and unshocked embryos was carried out as described on the FlyChip website [55]. Four independent samples were labeled and hybridized to the cDNA arrays. Data processing, normalization and statistical analysis were as described above for the ChIP-array studies.

### Binding site distribution

The 1 kb of sequence immediately upstream of all transcripts of genes within Release 1 of the *Drosophila *Gene Collection (DGC1) was obtained by filtering the corresponding sequences recovered from Ensembl version 24.3b.1 (BDGP Release 3.1) via EnsMart against a list of DGC1 genes obtained from FlyBase. This subset was then searched for partial matches (mismatches <2) to an Hsf consensus sequence (GAAnnTTCnnGAA [43]) on both strands and statistics on the total number of hits for each of the DGC1 fragments calculated with custom-written BioJava-based software. The distribution of hits against the 137 Hsf-binding genes and the remaining non-Hsf-binding genes were then plotted against increasing hit counts.

### Data availability

Raw and processed microarray data are available from the national Center for Biotechnology Information (NCBI) Gene Expression Omnibus site [58] with the following series accession numbers: genome tile arrays, GSE2423; cDNA arrays, GSE2398.

## Additional data files

The following additional data are available with the online version of this paper. Additional data file 1 contains a tab-delimited table containing the ratios and CyberT statistics for the genome tile array. Additional data file 2 contains a tab-delimited file of data from the cDNA arrays. Additional data file 3 contains compiled data for the top 188 cDNA clones. Additional data file 4 contains sequences of the primer pairs used to amplify each of the fragments on the genome tile array.

## Supplementary Material

Additional Data File 1 Tab delimited table containing the ratios and CyberT statistics for the genome tile array. For each fragment (Clone_ID) the VSN-normal*ized *ratio (average of the dye swaps) of Hsf-enriched v control for each independent immunopurification is given. The CyberT statistics are: #obs = number of measurements accepted, Mn = mean ratio, SD = standard deviation of the mean, t = t-test statistic calculated using the standard deviation,*p *= *p*-value associated with the t-test. Click here for file

Additional Data File 2 Tab-delimited file of data from the cDNA arrays. The average ratio for each of the 7 independent immunopurifications from 3 separate chromatin preparations is given (N = number of technical replicates used to calculate the average). For each clone the FlyBase ID, gene symbol and BDGP clone number is given. The headers indicate the chromatin batch (A, B and C) and immunopurification (1-4). Click here for file

Additional Data File 3 Compiled data for the top 188 cDNA clones. For each gene the Fbgn and FlyBase symbol is given followed by the summarized CyberT statistics, mean ratio (Mn) and *p *value. The remaining data are: Custom Affymetrix array (Affy), expression ratio and *p*-value from cDNA arrays (cDNA and cDNA P), GAGA-factor DAM-ID experiment (GAGA, GAGA P), number of predicted Hsf sites in the 1kb upstream fragment, predicted cytological location from FlyBase, yeast homologue, expression and percentile ranking from Hahn *et al. *[28] (MaxExp, Non-HS, HS), *p *value and ratios from Lee *et al*. [27] and the blast score of *Drosophila *vs yeast protein matches. Click here for file

Additional Data File 4 Sequences of the primer pairs used to amplify each of the fragments on the genome tile array. Clone_ID - identifier as in Additional data file 1, 5' and 3' - primer sequences, size - PCR amplicon size. Click here for file

## Figures and Tables

**Table 1:** Enrichment of HSE with anti-Hsf ChIP as measured by quantitative real-time PCR

Hsp	Primer pairs used	Fold enrichment
Hsp26	5' HSE	110
*Hsp26*	3' UTR	< 0.1
*Hsp70A*	5' HSE	103
*Hsp70A*	3' UTR	3.5

DNA was analyzed by quantitative real-time PCR as described in Materials and methods using primer pairs specific for the 5' HSE and 3' UTR regions of *Hsp26 *and *Hsp70A*. Fold enrichment is based on the comparison between amplifications with DNA from ChIP using anti-Hsf or preimmune antiseum.

**Table 2:** Top 50 cDNA clones identified by anti-HSF ChIP on cDNA arrays

FlyBase gene	Mean ratio	*p*-value	Gene chip	cDNA	DAM GAGA	GAGA *p*-value	HSF sites	Cytology
*CG32041*	3.043	2.02E-05	-	**15**	**1.305**	1.25E-05	5	**67B1**
*CG1416*	2.793	2.19E-04	-	**2.4**	-0.024	8.44E-01	1	**40A2**
*CG9705*	2.674	4.91E-05	-	1.5	0.118	4.86E-01	8	**73C4**
*CG3428*	2.428	6.53E-05	-	**2.1**	-0.086	4.76E-01	3	**67B8**
*DnaJ-1*	2.375	5.25E-04	6.13	**4.4**	**0.489**	4.23E-03	1	64E5
*FKBP59*	2.321	3.88E-04	-	2.4	-0.047	6.19E-01	1	30E1
*CG1553*	2.179	3.11E-05	-	**2.4**	0.368	1.47E-02	2	**43E17**
*Hsc70Cb*	2.164	2.90E-04	-	**2.5**	0.189	2.89E-01	1	**70C15**
*Taf7*	2.128	3.06E-06	-	1.2	**0.462**	5.95E-03	1	**84E5**
*CG10286*	2.128	6.95E-06	-	1.2	0.226	1.01E-01	5	83E4
*CG2182*	2.080	1.85E-05	-	1.1	0.188	1.26E-01	5	83B8
*MESR6*	2.079	9.11E-06	-	1.6	0.104	3.14E-01	4	**75F7**
*Fer1HCH*	1.986	4.23E-05	-1.09	0	**1.793**	6.62E-06	6	**99F2**
*CG8258*	1.962	3.83E-05	-	1.4	0.215	1.32E-01	4	**44F5**
*CG11455*	1.954	1.47E-03	-	0	0.100	5.55E-01	4	**21B1**
*EP2237*	1.928	3.25E-04	-	**1.4**	0.258	4.45E-02	0	21D6
*alphaCop*	1.926	4.35E-04	-	-0.7	0.820	5.90E-01	5	62A9
*Trap36*	1.919	1.58E-04	-	**-2**	-0.208	7.55E-02	2	65F2
*Sir2*	1.917	1.16E-04	-	1.4	0.280	4.32E-02	9	**34A7**
*CG11791*	1.906	5.08E-06	-	1.3	**0.490**	4.34E-03	3	96B19
*CG32649*	1.836	7.90E-04	-	2	0.064	5.98E-01	5	11D1
*l(1)G0331*	1.833	1.13E-04	-	1.3	0.143	1.77E-01	3	7B1
*Cyp1*	1.805	9.67E-05	-1.13	0	0.109	3.59E-01	1	14B12
*RNaseX25*	1.803	6.23E-05	-	1.1	-0.310	1.87E-02	2	66A21
*l(2)08717*	1.794	7.56E-04	-	0	**1.624**	1.49E-07	2	55F3
*CG10576*	1.724	2.14E-04	-	1.3	-0.329	4.11E-03	6	64E6
*Xbp1*	1.710	2.23E-04	-	1.5	0.108	3.20E-01	6	**57C3**
*Pgi*	1.708	1.65E-03	2.01	1.4	-0.011	9.08E-01	2	**44F6**
*Hsc70-5*	1.686	1.76E-04	1.44	2	0.019	8.58E-01	3	**50E6**
*sgl*	1.667	1.74E-07	1.84	1.6	0.172	2.51E-01	0	64D4
*Hsp23*	1.665	7.44E-04	10.11	**21**	**0.786**	2.56E-04	14	**67B1**
*Arf79F*	1.651	7.42E-04	1.08	0	0.277	7.76E-02	2	80B2
*CG8297*	1.623	1.95E-03	-	**1.9**	-0.208	1.77E-01	5	52D2
*dmt*	1.623	1.39E-03	-	1.2	-0.175	1.19E-01	2	**85E5**
*l(1)G0022*	1.591	1.16E-03	-	1.2	-0.110	3.57E-01	3	**13E14**
*CG7945*	1.581	9.89E-05	-	**-2.6**	0.034	7.40E-01	5	71D4
*CG31536*	1.579	1.06E-04	-	0	-0.045	7.04E-01	1	82E2
*Hsp27*	1.568	6.82E-04	12.42	**32**	**1.001**	4.92E-05	9	**67B1**
*Lrr47*	1.560	9.19E-04	-	1.1	-0.252	1.77E-02	1	**50E6**
*CG1103*	1.551	7.79E-04	-	-1.1	-0.310	1.16E-02	5	82A4
*CG10600*	1.539	9.21E-05	-	1.2	-0.145	1.51E-01	5	**37B1**
*CG10973*	1.532	7.99E-03	-	2	-0.148	1.52E-01	4	69E1
*CG12744*	1.496	5.10E-03	-	0	**0.693**	2.52E-03	3	46C1
*sra*	1.476	1.79E-04	-	**2.2**	-0.110	3.25E-01	6	89B12
*Rpn6*	1.469	8.39E-05	-	1.4	-0.237	4.20E-02	3	51C1-2
*CG3488*	1.466	8.40E-04	3.2	1.3	-0.023	9.13E-01	3	23D4
*sktl*	1.462	2.79E-03	1.14	1.1	-0.090	4.45E-01	5	57B3
*Actr13E*	1.447	1.04E-03	-1.27	-1.1	-0.288	2.35E-02	6	**13E12**
*CG17294*	1.447	1.81E-03	-	1.4	-0.241	2.30E-02	7	29B3
*CG33111*	1.426	1.27E-04	-	0	NA	NA	9	95B7

The FlyBase gene symbol, corresponding to the cDNA clone on the array, is given along with the mean asinh ratio and *p*-values derived from Cyber-T. Expression data is given from custom Affymetrix GeneChips and from the cDNA arrays with RNA extracted from heat-shocked embryos; bold indicates significant expression (*p *better than 10^-3^). The mean ratios and *p*-values from a GAGA-factor DamID experiment are listed for each gene; bold indicates significant ratios. Hsf sites indicates the number of predicted Hsf sites found 1 kb upstream of each gene and the column heading cytology indicates the predicted cytological location; matches with the polytene chromosome studies are in bold. See text for details. The full list of 188 genes with associated data is given in Additional data file 3.

**Table 3:** Genes binding Hsf in both *Drosophila *and *S. cerevisiae*

FlyBase	Fly ratio	Yeast	Yeast express	NO-HS	HS	Lee ratio	Lee *p*	BLAST	Yeast GO function
Acon	0.968	ACO1	2.876	0.833	0.516	1.86	6.90E-02	4.00E-274	Aconitate hydratase
*Hsc70-5*	1.686	SSC1	1.98	0.999	0.999	7.05	1.50E-04	6.60E-201	Protein transporter*
*Hsp60*	1.039	HSP60	2.608	0.997	0.997	2.33	1.50E-02	1.00E-177	Single-stranded DNA binding*
*Cctgamma*	1.275	CCT3	-0.28	0.646	0.343	1.85	7.80E-02	4.00E-162	Unfolded protein binding*
*l(1)G0022*	1.591	CCT6	0.063	0.714	0.921	1.73	8.60E-02	1.00E-136	Unfolded protein binding*
*Hsc70Cb*	2.164	SSE1	1.771	0.998	0.999	4.48	1.50E-03	1.70E-115	Unfolded protein binding*
*CG8863*	0.818	YDJ1	1.529	0.999	0.997	6.62	2.40E-04	2.00E-74	Chaperone regulator*
*Cyp1*	1.805	CPR1	1.251	0.996	0.995	10.83	3.40E-05	3.00E-65	Peptidyl-prolyl *cis*-*trans *isomerase
*CG8258*	1.962	CCT4	NA	0.385	0.055	1.85	7.80E-02	3.00E-55	Unfolded protein binding*
*CG2918*	1.100	SSA3	4.479	0.934	0.995	1.19	4.80E-01	7.00E-50	ATPase
*DnaJ-1*	2.375	SIS1	3.237	0.999	0.999	11.31	3.50E-05	7.00E-46	Unfolded protein binding*
*Rab35*	1.416	YPT32	0.338	0.269	0.343	1.98	4.30E-02	4.00E-40	GTPase activity
*sktl*	1.462	MSS4	1.042	0.712	0.879	1.91	4.80E-02	7.00E-35	Phosphatidylinositol kinase
*CG4800*	1.424	RBF18	0.03	0.872	0.964	2.13	1.50E-01	1.00E-33	Unknown
*Cyt-c-d*	0.932	CYC1	1.738	0.959	0.942	2.53	1.10E-02	4.70E-33	Electron carrier
*mbf1*	0.706	MBF1	NA	0.999	0.998	9.4	1.80E-04	2.00E-29	Transcription coactivator
*CG1416*	2.793	AHA1	3.288	0.999	0.999	9.14	5.60E-04	3.00E-28	Chaperone activator*
*CG4500*	1.37	FAA1	3.889	0.796	0.943	2.61	8.00E-03	9.00E-23	Fatty-acid-CoA ligase
*CG32920*	1.081	AHP1	1.547	0.983	0.984	3.81	1.10E-03	1.10E-19	Thioredoxin peroxidase
*SH3PX1*	0.877	SNX4	1.26	0.824	0.372	2.52	1.30E-02	6.30E-13	lipid binding
*CG10973*	1.532	FES1	3.541	0.997	0.993	NA	NA	1.10E-10	Adenyl-nucleotide exchange factor
*l(2)35Bg*	0.755	DRE2	0.304	0.843	0.832	3.36	3.70E-03	5.00E-10	Unknown
*CG12200*	0.786	CST9	1.022	0.346	0.467	1.69	9.40E-02	8.70E-10	DNA binding

The FlyBase gene ID is given, along with the average asinh ratio of Hsf enrichment. Yeast homologs are indicated by their *Saccharomyces *genome database (SGD) common names. The average heat-induced expression and ranking of Hsf binding in non-heat shocked (NO-HS) and heat shocked (HS) cells are from [28], the Lee ratio and Lee *p *are from [27]. BLAST scores are derived from searches at the SGD using the *Drosophila *sequences as probes. Gene Ontology (GO) functional classifications are for the yeast proteins; asterisks indicate stress-response proteins.

**Table 4:** Primers used in the standard PCR analysis

Gene	5' primer	3' primer	Product (bp)
CG3273	ACCTGGCGGAATATCACAGA	ACCCCAATGTCGGATGTAGA	421
*CG9746*	GCGAAAACCAATCGATGTTA	CGAAGCAAGATGACCTTTCC	403
*CG10077*	CGACCCAAAAACCAAAGTGT	GATATCGGTTTTCGCCTTCA	444
*CG11166*	GGCCTGCGAGGAAAAGTTAT	GTCGATCCCAACAGCTACAA	414
*CG12876*	TTTTTATTACTAACATGAACCGGTAA	GCCGTTGGTTTCTCCACTT	408
*CG33111*	TACGCAGCGAATATCGATTG	TTCTGCACGAGGGGTAGTCT	417
*CG33144*	CCCAATTGGAAATGAGTGCT	GAATTTCCTAAATTTTGCAAGGA	441
*dmt*	ACCATCCCCCGATCTCTAAG	GCAGGCAGGAAAATCACAAT	404
*EP2237*	GAAAAAGGCAAAGCCATTCA	CTCGGAAAAGATGGCAACAT	451
*MBF1*	CCAGATGGTTAAACGGCAAT	GGCTCAAGGAGCTACTGAAAAA	405
*veg*	AATTCTCGTTGCTCTCGAACT	TGGAGTTCTTCTTGGCCACT	409
